# Dr. Walker, on Thoracic Viscera, &c.

**Published:** 1810-01

**Authors:** 


### *To the Editors of the Medical and Physical Journal.*

GENTLEMEN,

SOME ideas lately occurring to me, on certain parts of the Economy of the Thoracic Viscera, I offer them to your notice, in the most hasty manner; for I know not that I had ever less leisure for preparing a paper of correct and cautious utterance.

It is well known, that the contents of the cranium swell gently under expiration, when the blood has not the most free and open passage through the lungs; that they are diminished under inspiration. While this regular alternation of pressure and its removal, may keep up a gentle and regular intestine motion throughout the sensorium, necessary for supporting its inconceivably exquisite functions, which a mere circulation of fluid could not effect; the whole mass of blood, in passing through the lungs, where it has to undergo important changes, may also be affected to the furtherance of these changes, by the alternation of elongation and shrinking, which all its conducting vessels are subjected to from respiration.

If the changes were, principally, as they are generally considered, only chemical, might not I, in support of the conjecture, just observe how instantaneously a crystallization sometimes takes place, in the laboratory, on the vessel where it is expected getting a trifling shake. Perhaps, however, while the origin of animal heat is, with so much ingenuity, referred chiefly to chemical agency, it may, in fact, principally have its source in that law of physiology, which requires inflammation in the reparation of parts. The reparation of parts is an incessant process throughout the whole animal system.

But to return from the digression.—If functions in the animal economy are accelerated by alternation of extra pressure and its removal on the blood vessels, which may possibly be the case, then, with what inconceivable rapidity must the change which the blood undergoes in its passage through the coronary vessels be effected; must the supplies of nourishment be laid hold of, through all its fibres, by this incessantly acting vital muscle!

Cheselden says, in his Anatomy, "Over the entrance of the auricles, in each ventricle, are placed valves to hinder a return of blood while the heart contracts. Those in the right ventricle are named, Tricuspides; those in the left, Mitrales. One of these last seems to do further service, vice, by covering the mouth of the aorta, while the ventricle fills; which suffering none of the blood to pass out of this ventricle into the aorta before the ventricle acts, it will be able to give greater force to the blood than it otherwise might have done, because a greater quantity of blood more fully distending the ventricle, and making the greater resistance, it will be capable of receiving the greater impelled force from the ventricle; and if the blood is no way hindered in the right ventricle from getting into the pulmonary artery, while the ventricle dilates, as it is in the left, the left then must be somewhat bigger than the right, if they both empty themselves in every systole."

The blood also, being impelled with less force into the lungs, at the time of systole, it may be said that they are hereby guarded from too great a continually iterated force, while, from the smallness of the pulmonary system of veins, they are guarded from being charged with too heavy a load.

Boerhaave, in his Institutiones Medicæ, says of the coronary vessels. "*Hæ arteriæ sunt in diastole, dum reliquæ corporis arteriæ in systole constituuntur.*"—"*Hæ venæ inaniuntur, dum reliquæ corporis venæ implentur.*"

Perhaps it is necessary, that the coronary artery conveying blood into the substance of the heart, should be guarded against the great impetus of its left ventricle.

"From under two of the semilunar valves of the aorta, which is, ere it leaves the heart, arise two branches (sometimes but one) which are bestowed on the heart, and are called Coronariæ Cordis."—*Cheselden's Anatomy.*

By the systole of the heart, the semilunar valves of the aorta are thrown open. They close up the orifice of the coronary artery, and help to prevent regurgitation from it, while the blood throughout the whole system of vessels, forming principally the substance of the heart, is under excessive pressure. The arterial systole commences at the heart. The valves are thrown down. The ventricles in a state of constriction, permit not the arterial systole to impel blood into their proper arteries; have thrown out the blood, by their constriction, from the coronary veins with rapidity; are holding their arterial blood under such pressure, as (with rapidity) to make it give up whatever nutriment it can afford them, to charge it with what is to be carried off through the right side of the heart to the lungs for removal. The ventricles slowly relaxing, the coronary artery opens, invites, and in a gentle current, receives from the never, during life, wholly exhausted aorta, the necessary quantity of blood to cause its own contraction; first at its orifice, then, progressively through its ramifications and into the coronary veins, to be again rapidly squeezed out, on the next systole, to the right auricle of the heart.

Respectfully,

J. W.

*Salisbury Court*, 21, *xij*, 1809.

